# Somatic piRNA and PIWI-mediated post-transcriptional gene regulation in stem cells and disease

**DOI:** 10.3389/fcell.2024.1495035

**Published:** 2024-12-09

**Authors:** Mahammed Zaid Patel, Yuguan Jiang, Pavan Kumar Kakumani

**Affiliations:** Department of Biochemistry, Memorial University of Newfoundland, St. John’s, NL, Canada

**Keywords:** piRNA, PIWI, mRNA, retrotransposons, gene regulation, stem cells, disease

## Abstract

PIWI-interacting RNAs (piRNAs) are small non-coding RNAs that bind to the PIWI subclass of the Argonaute protein family and are essential for maintaining germline integrity. Initially discovered in *Drosophila*, PIWI proteins safeguard piRNAs, forming ribonucleoprotein (RNP) complexes, crucial for regulating gene expression and genome stability, by suppressing transposable elements (TEs). Recent insights revealed that piRNAs and PIWI proteins, known for their roles in germline maintenance, significantly influence mRNA stability, translation and retrotransposon silencing in both stem cells and bodily tissues. In the current review, we explore the multifaceted roles of piRNAs and PIWI proteins in numerous biological contexts, emphasizing their involvement in stem cell maintenance, differentiation, and the development of human diseases. Additionally, we discussed the up-and-coming animal models, beyond the classical fruit fly and earthworm systems, for studying piRNA-PIWIs in self-renewal and cell differentiation. Further, our review offers new insights and discusses the emerging roles of piRNA-dependent and independent functions of PIWI proteins in the soma, especially the mRNA regulation at the post-transcriptional level, governing stem cell characteristics, tumor development, and cardiovascular and neurodegenerative diseases.

## 1 Introduction

### 1.1 PIWI proteins and piRNAs

**FIGURE 1:**
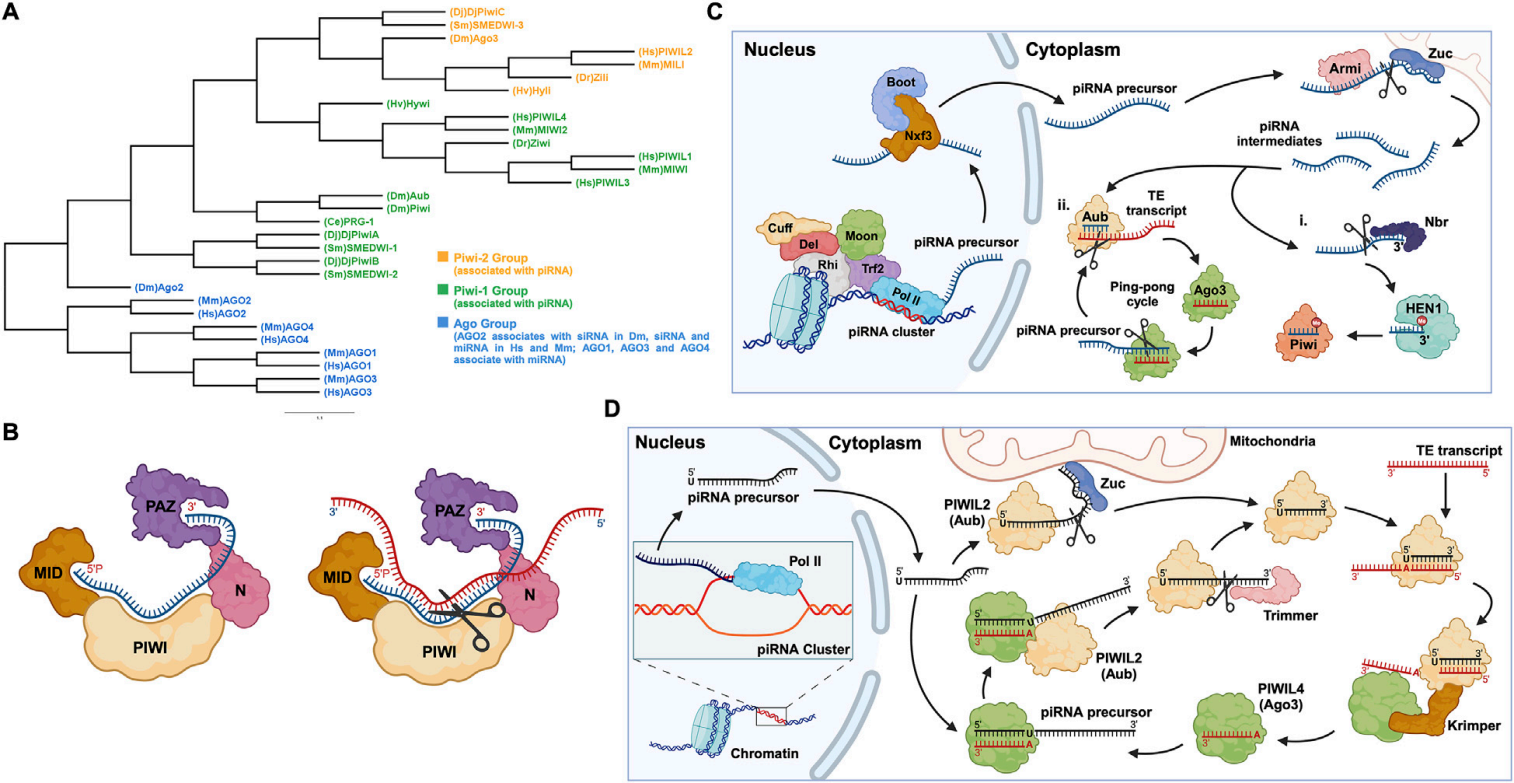
PIWI proteins and their functional domains in the piRNA-PIWI complex, and the overview of the piRNA pathway in *Drosophila*. **(A)** Phylogenetic tree of the Argonaute (AGO) protein family with each class of AGOs specific to the type of small non-coding RNAs. Hs: *Homo sapiens*, Mm: *Mus musculus*, Sm: *Schmidtea mediterranea*, Dr: *Danio rerio*, Hv: *Hydra vulgaris*, Dm: *Drosophila melanogaster*, Dj: *Dugesia japonica*, Ce: *Caenorhabditis elegans*. **(B)** (left) The MID domain of PIWI proteins binds to the 5′phosphate group of piRNA (shown in blue), and the PAZ domain binds to the 2′-O-methylated 3′end of piRNA (right). The target RNA is captured by piRNA through base-pair complementarity, and the PIWI domain, which possesses ribonuclease activity, cleaves the target RNA (shown in red). **(C)** The piRNA biogenesis in *Drosophila*: First, the piRNA cluster on the chromosome is transcribed by the RNA Pol II to produce piRNA precursors with the assistance of proteins, namely Rhino (Rhi), Cutoff (Cuff), Deadlock (Del), Trf2, and Moonshiner (Moon). The piRNA precursors are transported out of the nucleus by proteins such as Nxf3 and Bootlegger (Boot), and their secondary structure is removed by Armitage (Armi), and cut by Zucchini to form piRNA intermediates. Following, some piRNA intermediates, after being trimmed by Nibbler (Nbr) and methylated at the 3′end by Hen1, bind to Piwi (PIWIL1 homolog) to form a Piwi-piRNA complex (i). Other piRNA intermediates bind to Aub (PIWIL2 homolog) and then enter the ping-pong cycle dominated by Aub and Ago3 (PIWI4 homolog) (ii). **(D)** The ping-pong cycle, widely conserved in zebrafish, mice, humans, and other animals, silences target RNAs (such as TE transcripts) and amplifies piRNAs. The initial piRNA precursor, transcribed by RNA Pol II is exported from the nucleus to the cell cytoplasm, where it is processed into a mature form. After binding to the Aub protein, the mature piRNA cleaves the target TE transcript. The fragments of the cleaved transcript, bound by the Ago3 protein, act as a template and facilitate the processing of the piRNA precursor. The processed piRNA precursor, bound by the Aub protein and cleaved with the help of the protein Trimmer, enters the cycle to continue the silencing of complementary transcripts and the generation of piRNAs.

PIWI-interacting RNAs, called piRNAs, are single-stranded small RNAs with a size of 24–31 nucleotides (Iwasaki et al., 2015). They are bound by a special class of proteins, namely, PIWI - an abbreviation of P-element Induced Wimpy testis in *Drosophila*. (Lin and Spradling, 1997; Sohn and Oh, 2023). The *piwi* gene was first identified to be involved in germline stem cell division in *Drosophila* gonads, in both ovaries and testis (Lin and Spradling, 1997). Subsequently, piRNAs were discovered in mouse testis in 2006 and were defined as small non-coding RNAs that specifically interact with PIWI proteins (Aravin et al., 2006; Girard et al., 2006). For distinction, the name Piwi refers to one of the specific proteins in *Drosophila*, while PIWI refers to the protein subfamily (Ross et al., 2014). Additionally, PIWIs belong to the Argonaute proteins family, which encompasses another branch containing Ago proteins that bind to a different class of small RNAs, specifically, microRNAs (miRNAs) and small interfering RNAs (siRNAs) (Wang X. et al., 2023) (Figure 1A). Like the fellow members of Argonaute protein family, PIWIs have a bilobal structure, with lobes at both the N- and C-terminus binding to the 5′ and 3′ ends of piRNAs, like a pocket, protecting the piRNA (Matsumoto et al., 2016; Dayeh et al., 2018). The PIWI proteins are well conserved among different classes of animal species (Kim et al., 2018; Huang and Wong, 2021) (Table 1). The PIWI proteins consist of four main domains, namely, PIWI-Argonaute-Zwille (PAZ), N-terminal (N), PIWI, and middle (MID). The PAZ domain recognizes and binds to the 2′-O-methylated 3′end of piRNA, while the MID domain binds to the 5′uridine end, and the PIWI domain acts as an RNase H endonuclease to cleave the target transcript captured by piRNA through complementary base pairing (Litwin et al., 2017) (Figure 1B).

**TABLE 1:** Nomenclature of the PIWI proteins in different organisms.

Species	PIWI name	Alias	References
*Homo sapiens*	HIWI	PIWIL1	Loubalova et al. (2023)
	HILI	PIWIL2	
	HIWI3	PIWIL3	
	HIWI2	PIWIL4	
*Drosophila melanogaster*	Piwi	N.D.	Huang and Wong (2021), Loubalova et al. (2023)
	Aub		
	Ago3		
*Mus musculus*	MIWI	PIWIL1	Cheng et al. (2014), Loubalova et al. (2023)
	MILI	PIWIL2	
	MIWI2	PIWIL4	
*Caenorhabditis elegans*	PRG-1	-	Kim et al. (2018)
*Planarian*	SMEDWI-1	PIWI-1, PIWI-A	Kashima et al. (2020), Li et al. (2021)
	SMEDWI-2	PIWI-2, PIWI-B	
	SMEDWI-3	PIWI-3, PIWI-C	
*Dugesia japonica*	DjpiwiA	N.D.	Shibata et al. (2016)
	DjpiwiB		
*Hydra vulgaris*	Hywi		Nishimiya-Fujisawa and Kobayashi (2018), Teefy et al. (2020)
	Hyli		
*Hydractinia symbiolongicarpus*	Piwi1		Varley et al. (2022)
*Hydractinia echinata*	Piwi1		Bradshaw et al. (2015)
*Notospermus geniculatus*	Ng-piwi1		Xu and Sun (2020)
	Ng-piwi2		
	Ng-piwi3		
*Lineus sanguineus*	Ls-piwi1		Xu and Sun (2020)
	Ls-piwi2		
	Ls-piwi3		
*Hofstenia miamia*	piwi-1		Hulett et al. (2022)
*Mnemiopsis*	MlPiwi1		Reitzel et al. (2016)
*Parasteatoda tepidariorum*	Pt-piwi		Schwager et al. (2015)

N.D., not determined.

### 1.2 piRNA biogenesis and the formation of piRNA-PIWI complex

piRNAs are generated from specific gene loci called piRNA clusters (Parhad and Theurkauf, 2019). In the cell nucleus, piRNA clusters are transcribed by RNA Polymerase II (RNA Pol II), into piRNA precursors, then these transcripts are exported to the cytoplasm through nuclear pores and are processed by enzymes such as Zucchini to form smaller piRNA fragments called piRNA intermediates (Huang and Wong, 2021; Ramat and Simonelig, 2021; Allikka Parambil et al., 2024). Most of these fragments are bound by the homologous protein PIWIL2, enter the ping-pong cycle (a process described in detail later on, in this review), or are methylated at the 3′end by the methyltransferase Hen1, forming a mature piRNA complex with the PIWIL1 homolog (Gainetdinov et al., 2018; Huang and Wong, 2021, p. 20121) (Figure 1C). Here, it is worth mentioning that, unlike the other two well-known small RNAs (miRNA and siRNA), the precursor of piRNA is a single-stranded RNA (ssRNA) rather than double-stranded RNA (dsRNA), and thus the production of mature piRNAs is independent of the enzyme Dicer, an RNase III endoribonuclease (Iwasaki et al., 2015).

### 1.3 TE silencing - a basic function of piRNA-PIWI complexes

In humans, transposable elements (TEs), also called “jumping genes”, and their derivatives account for 44%–48% of the genome (Autio et al., 2021; Chénais, 2022). TEs are inserted into protein-coding genes in numerous ways, causing genetic mutations and destabilizing the gene structure (Parhad and Theurkauf, 2019). Briefly, the mature piRNA complex which is exported to the nucleus from the cytoplasm, binds to complementary sites in nascent target TE transcripts and silences their expression, thus protecting the cell (Parhad and Theurkauf, 2019; Ramat and Simonelig, 2021). Additionally, piRNA complexes can silence TE transcripts in the cytoplasm and can activate, protect, and localize a wider range of other messenger RNAs (mRNAs) (Ramat and Simonelig, 2021). Most piRNAs and PIWIs gather in the “Nuage” region of the perinuclear cytoplasm, where they undergo a ping-pong cycle (Pek et al., 2012; Ge et al., 2019). The ping-pong cycle is an essential mechanism widely observed in flies, fish, and mammals, playing a critical role in piRNA amplification and the silencing of transposon transcripts (Czech and Hannon, 2016; Huang and Wong, 2021). Initially, researchers found that, in *Drosophila* germ cells, the 10 nucleotides at the 5′end of piRNAs that bind to Aub and Ago3, respectively, showed strong complementarity, indicating that these two classes of piRNAs are likely to interact with each other, which was later defined as the “ping-pong signature” (Brennecke et al., 2007; Li et al., 2009; Rojas-Ríos and Simonelig, 2018). For instance, during the ping-pong cycle in *Drosophila* germ cells, the piRNA-Aub complex binds and cleaves the target TE transcript (Rojas-Ríos and Simonelig, 2018). The cleaved fragment of the transcript with the exposed 3′ end is loaded into the Ago3 protein with the help of the Tudor-domain protein Krimper (Czech and Hannon, 2016). The Ago3 protein, guided by the RNA fragment, forms a complex with the piRNA precursor. Subsequently, the precursor is cleaved, and the fragment with the exposed 3′ end is loaded into the Aub protein, forming a new piRNA-Aub complex, aided by the Trimmer proteins (Czech and Hannon, 2016) (Figure 1D). The ping-pong cycle is conserved in other animal species, such as mouse, where the counterparts of Aub and Ago3 are MILI and MIWI2, respectively (Ernst et al., 2017; Huang and Wong, 2021), and the auxiliary proteins involved in this cycle are yet to be confirmed.

### 1.4 Functional implications of piRNAs and PIWIs in the soma and human diseases

Although initial studies in *Drosophila* led the researchers to believe that the piRNA pathway was limited to the germ line (Malone et al., 2009), recent studies have shown that piRNAs also function in mammalian and non-mammalian somatic tissues and stem cells. Further studies have discovered that depending on the context, for instance, in cancer cells, in addition to the piRNA-PIWI functional complex, piRNA and PIWIs can work independently of each other to control the expression of specific genes (Zhang et al., 2023).

In the current review, we explored the roles of piRNAs and PIWI proteins in stem cells other than germline, including Adult Stem Cells (ASCs), Embryonic Stem Cells (ESCs), Induced Pluripotent Stem Cells (iPSCs), etc. Additionally, we discussed the emerging roles of piRNA and PIWI proteins, both dependent and independent of each other, in regulating the expression of TE transcripts and mRNAs along with their translation in somatic cells and the development of numerous human diseases.

## 2 PIWIs in adult/somatic/embryonic stem cells

**FIGURE 2:**
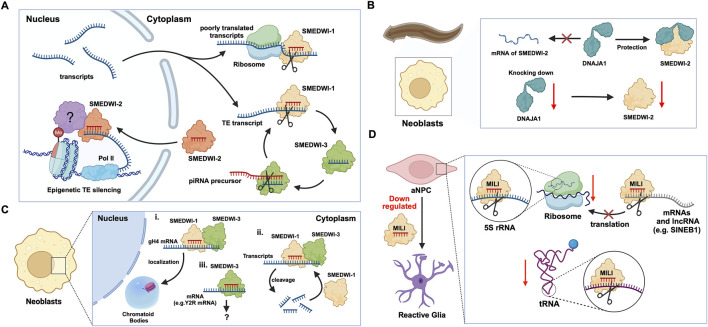
The functions of PIWI proteins in planarian adult pluripotent stem cells (aPSCs) and mouse adult neural progenitor cells (aNPCs). **(A)** In planarian aPSCs (neoblasts), TEs are mainly silenced in the nucleus. The SMEDWI-2 complex binds to TEs that are being transcribed, induces H3K9 methylation, and prevents further transcription. For other TE transcripts that successfully enter the cytoplasm, the SMEDWI-1 complex silences them through the ping-pong cycle. In addition, poorly translated transcripts are recognized and silenced by the SMEDWI-1 complex, facilitated by its interaction with the small ribosomal subunit. **(B)** Knockdown of DNAJA1 in neoblasts reduced the abundance of SMEDWI-2 protein but had little effect on the expression levels of SMEDWI-2 transcripts. **(C)** The effects of SMEDWI-1 and SMEDWI-3 on mRNA in neoblasts: (i) SMEDWI-1 and SMEDWI-3 assist germinal histone H4 (gH4) transcripts in localizing to chromatoid bodies. (ii) SMEDWI-1 and SMEDWI-3, guided by the piRNAs, cleave mRNAs that code for all the histone proteins, Traf-6, and Npk1-like proteins. The cleaved mRNAs become templates for the newly generated piRNA-SMEDWI-1 complexes. (iii) The piRNA-SMEDWI-3 complexes bind other mRNAs, such as Y2R mRNA. However, no changes were observed in the mRNA levels, and this association’s outcome is yet to be determined (denoted as ?). **(D)** The piRNA-MILI complex is involved in the preservation of mouse aNPC pluripotency. (left) Artificial knockout of MILI causes aNPCs to differentiate into unhealthy astrocytes, also referred to, as reactive glia. (right) The piRNA-MILI complex inhibits protein synthesis in aNPCs by silencing tRNA, 5S rRNA, SINEB1 and mRNAs encoding ribosomal proteins, thereby slowing down their differentiation.

Planaria is a promising animal model for studying adult stem cells and cell regeneration. Planarian adult pluripotent stem cells (aPSCs, also known as neoblasts) express three known PIWI protein homologs, PIWI-1 (SMEDWI-1), PIWI-2 (SMEDWI-2), and PIWI-3 (SMEDWI-3) (Shibata et al., 2016; Kashima et al., 2020; Li et al., 2021). Knocking down the three PIWI proteins revealed that SMEDWI-2 and SMEDWI-3 are necessary for planarian regeneration. The absence of the two PIWIs led to defects in the regenerative capacity of the planaria, and even cell death (Dattani et al., 2019), while the deficiency of SMEDWI-1 resulted in a decrease in the number of stem cells and a delay in the expansion of new cells (Allikka Parambil et al., 2024). Among the three PIWIs, SMEDWI-2 silences transposons at the epigenetic level in the nucleus (Dattani et al., 2019), and is required for homeostasis and regeneration of planarian stem cells (Almazan et al., 2018). Whereas transposons that escape nuclear silencing by SMEDWI-2 are eliminated by SMEDWI-1 and SMEDWI-3, after entering the cytoplasm, a process known as post-transcriptional silencing (Shibata et al., 2016; Li et al., 2021) (Figure 2A). SMEDWI-1 and SMEDWI-3 are the counterparts of *Drosophila* cytoplasmic PIWI proteins, Aub and Ago3 respectively (Sahu et al., 2017). These two PIWI proteins participate in the “ping-pong cycle” in planarian cell cytoplasm, to silence target TE transcripts and produce more piRNAs through amplification (Sahu et al., 2017; Dattani et al., 2019). As for SMEDWI-1, contrary to the earlier assumptions on its involvement as dispensable, recent studies discovered that SMEDWI-1 produces and stores piRNAs in the cell cytoplasm, binds to ribosomes, monitors poorly translated transcripts, and silences them during the pioneer round of translation (Allikka Parambil et al., 2024). Similarly, organisms such as sponges, jellyfish and planaria, carry totipotent somatic stem cells, called neoblasts (Sturm et al., 2017). Studies on the planarian *Dugesia japonica* revealed that DjpiwiB is essential for the differentiation of neoblasts into specialized somatic cells. Loss of DjpiwiB expression results in significant defects in the regeneration ability of neoblasts (Shibata et al., 2016; Rojas-Ríos and Simonelig, 2018) (Tables 1, 2). Additionally, investigations in the planarian *Schmidtea mediterranea* have shown that the PIWI-2 homologue SMEDWI-2 interacts with the heat shock protein DNAJA1, and both proteins are enriched in the planarian adult stem cells (Fernández-Taboada et al., 2011; Wang et al., 2019). Interestingly, the loss of DNAJA1 led to significant decrease in the protein levels of SMEDWI-2 in the cell cytoplasm, while the levels of SMEDWI-2 mRNA were unaltered, suggesting that DNAJA1 is likely to bind to SMEDWI-2 and maintain the stability of the latter (Wang et al., 2019) (Figure 2B). Also, DNAJA1 protects both SMEDWI-2 and piRNAs from factors like cellular environmental stress, and is essential for the maintenance of adult stem cells (Wang et al., 2019). Furthermore, studies on endodermal and ectodermal epithelial stem cells (eSCs) and i-cells of the Cnidarian *Hydra* have shown that it expresses two PIWI proteins, Hywi and Hyli (Nishimiya-Fujisawa and Kobayashi, 2018; Teefy et al., 2020) (Table 2). RNA immunoprecipitation studies showed that Hywi binds piRNAs and localizes to complementary sites on TE transcripts (Teefy et al., 2020). Degradome-Seq of RNA ends revealed that the 5′end of the piRNA bound to Hywi overlapped with the 5′end of the piRNA bound to Hyli by 10 bp (Juliano et al., 2014), which, together with the upregulation of TE expression brought about by the knockdown of Hywi demonstrated that Hywi and Hyli participate in the classic ping-pong cycle of the piRNA pathway to silence TE transcripts (Teefy et al., 2020). In addition, researchers have found that PIWI homolog, Hiwi, is expressed in human hematopoietic stem cells (HSCs) with CD34, and proved that it is likely to be a negative developmental regulator (Sharma et al., 2001; Rojas-Ríos and Simonelig, 2018). Subsequent studies found MIWI2 in mouse primitive hematopoietic cells and demonstrated that the loss of MIWI2 induces the differentiation of hematopoietic cells, but has little effect on the overall function of the hematopoiesis (Jacobs et al., 2013; Nolde et al., 2013; Rojas-Ríos and Simonelig, 2018).

**TABLE 2:** piRNAs, PIWIs and their roles in adult stem cells.

PIWI proteins or piRNAs	Species	Cell type	Function	References
SMEDWI-1	*Planarian*	Neoblasts	Post-transcriptional silencing of TEs through ping-pong cycle Silencing of poorly translated mRNAs Localizing germinal histone H4 mRNA to chromatoid bodies Degradation of transcripts that code for all the 5 histone proteins	Rouhana et al. (2014), Shibata et al. (2016), Li et al. (2021), Allikka Parambil et al. (2024)
SMEDWI-2	*Planarian*	Neoblasts	Transcriptional silencing of TEs	Dattani et al. (2019)
SMEDWI-3	*Planarian*	Neoblasts	Post-transcriptional silencing of TEs through ping-pong cycle Localizing germinal histone H4 mRNA to chromatoid bodies Degradation of transcripts that code for all the 5 histone proteins Degradation of other mRNAs	Rouhana et al. (2014), Shibata et al. (2016), Kim et al. (2019), Li et al. (2021)
DjpiwiB	*Dugesia japonica*	Neoblasts	Promotes neoblast differentiation	Shibata et al. (2016)
SMEDWI-1	*Schmidtea mediterranea*	Neoblasts	Silences RNAs, snRNAs, and pseudogene mRNAs Interacts with eIF4E, and participates in the pioneer round of translation	Choe et al. (2014), Allikka Parambil et al. (2024)
SMEDWI-2	*Schmidtea mediterranea*	Neoblasts	Protects DNAJA1 and maintains cell stability	Wang et al. (2019)
Hywi	*Hydra vulgaris*	eSCs, ISCs	Post-transcriptional silencing of TEs through ping-pong cycle	Teefy et al. (2020)
Hyli	*Hydra vulgaris*	eSCs, ISCs		Teefy et al. (2020)
Aub	*Drosophila*	ESCs	Post-transcriptional silencing of through ping-pong cycle Binds to Wispy and activates translation of mRNAs	Ramat et al. (2020), Huang and Wong (2021)
Ago	*Drosophila*	ESCs	Post-transcriptional silencing of TEs through ping-pong cycle	Huang and Wong (2021)
Piwi	*Drosophila*	ESCs	Post-transcriptional silencing of TEs	Huang and Wong (2021)
Piwi	*Drosophila*	ISCs	Silences multiple transcripts, such as Ty3 retrotransposon, mdg1, and hetA	Sousa-Victor et al. (2017)
HILI	*Homo sapiens*	hESCs	Promotes stem cell differentiation into mesoderm and further to contractile cardiomyocytes Participates in the regulation of multiple mRNAs	Vella et al. (2016), Liu et al. (2017), La Greca et al. (2020)
HIWI2	*Homo sapiens*	hESCs		Vella et al. (2016), Liu et al. (2017), La Greca et al. (2020)
MILI	*Mus musculus*	aNPCs	Maintains cell pluripotency Silences mRNAs encoding ribosomal proteins, 5S rRNA, and tRNAs	Gasperini et al. (2020), Gasperini et al. (2023)
Piwi1	*Hydractinia echinata*	i-cells	Protects stem cell stability and maintains cell stemness	Bradshaw et al. (2015)
Pt-piwi	*Parasteatoda tepidariorum*	ESCs	Participates in mitosis	Rui et al. (2020)
piR-63049	*Rattus*	BMSCs	Maintains the stemness of BMSCs	Chen et al. (2021)
piR-36741	*Homo sapiens*	BMSCs	Promotes BMSC differentiation into osteoblasts	Liu et al. (2021)
piR-007832 and piR-015026	*Homo sapiens*	BMMSCs	Involved in osteogenic differentiation	Jiang et al. (2019)
piR-020814 and piR-002732	*Homo sapiens*	BMMSCs	Communicates with hematopoietic stem cells via extracellular vesicles	Batsali et al. (2020), Wang et al. (2020)
piR-mmu-64162	*Mus musculus*	iPSCs	Regulates cell senescence and reprogramming processes in cellular pluripotency	Zhu et al. (2019)
piR-mmu-141534 and piR-mmu-429488	*Mus musculus*	NSCs	Carried by exosomes/microvesicles (Ex/Mv), with antiviral effects	Yu et al. (2020)

### 2.1 piRNA-PIWI-mediated regulation of mRNAs

**FIGURE 3:**
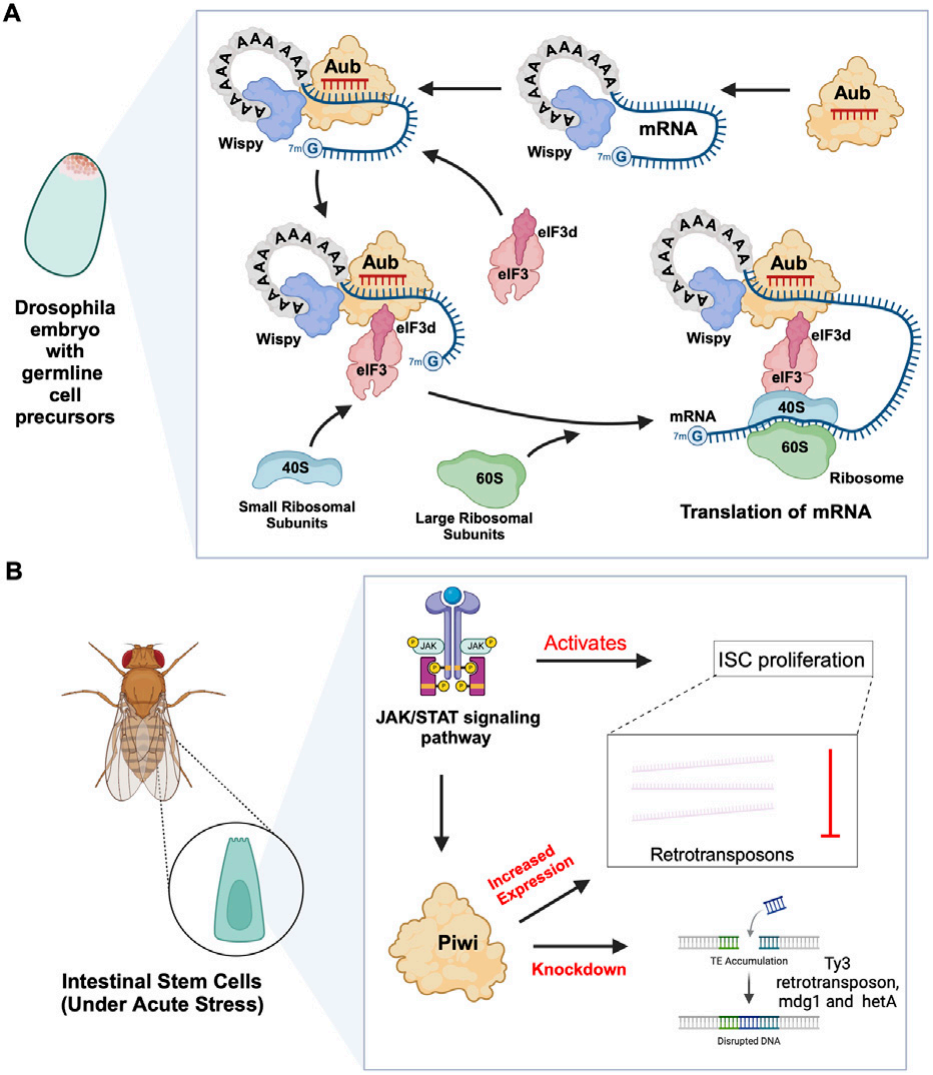
piRNA-PIWI-mediated mRNA regulation in *Drosophila* adult stem cells. **(A)** Aub activating mRNA translation in the early *Drosophila* embryo: The piRNA-Aub complex binds to complementary regions within mRNAs bound by the poly(A)-binding protein Wispy. When the mRNA needs to be activated, the Aub complex interacts with Wispy, and recruits eIF3d subunit, which is part of eIF3 complex, along with the 40S ribosomal subunit, thereby initiating the process of translation. **(B)** Role of JAK/STAT pathway and PIWI proteins in *Drosophila* ISCs: The JAK/STAT pathway activates ISC proliferation and induces the expression of *Drosophila* Piwi protein, which silences transposons and ensures gene stability. Knocking down Piwi led to the accumulation of multiple TE transcripts, including Ty3 retrotransposon, mdg1 and hetA.

In planarian neoblasts, SMEDWI-1 and SMEDWI-3 were reported to be crucial for the localization of germinal histone H4 (gH4) mRNA to chromatoid bodies (CB), a type of ribonucleoprotein (RNP) granules located in the perinuclear cytoplasm, and these PIWIs are indispensable for the formation of CBs(Rouhana et al., 2014). Also, recent studies have found that the piRNA- SMEDWI-1 complex binds to and regulates non-polyadenylated mRNAs, including stem-loop transcripts of all five histones during the S phase of the cell cycle (Allikka Parambil et al., 2024). Interestingly, during the differentiation of neoblasts, both CB and neoblast histone transcripts are degraded. More than 50 piRNAs which match the coding sequences of the transcripts of five histones respectfully have been identified. These piRNAs are likely to be the derivatives of a cleaved histone mRNA, and may therefore serve as potential candidates for guiding PIWI cleavage (Rouhana et al., 2014). Yet, it remains to be elucidated which specific PIWI proteins are involved in the degradation of histone transcripts. Additional work has demonstrated that SMEDWI-3 is involved in the degradation of a large number of mRNAs, including the transcripts of *Traf-6* and *Npk1*-like genes, in neoblasts. *Traf-6* is closely linked to the differentiation and proliferation of planarian stem cells, while the *Npk1-*like gene has not been studied in animals yet. At the same time, for other mRNAs, such as the transcripts of *Y2R* and *TEX2*, the piRNA-SMEDWI-3 complex binds to them but does not yield any change in their abundance, suggesting a translational control of these transcripts (Figure 2C; Table 2) or the RNA stabilization by the bound PIWIs, to maintain their function in the regulation of cell cycle (Kim et al., 2019). Likewise, in human embryonic stem cell (hESC) lines (H1, H9), during the differentiation of these pluripotent cells, the expression of HILI mRNA was significantly downregulated, while that of HIWI2 was upregulated (Liu et al., 2017; La Greca et al., 2020). In addition, small RNA-seq data showed that multiple piRNAs, including piR-4403262 and piR-4424378, may be involved in the regulation of numerous mRNAs in cardiomyocytes, including transcripts of *MALAT1*, *TTN*, and *PLN* (La Greca et al., 2020). Additionally, studies have shown that piRNAs promote the differentiation of cardiac progenitor cells and the regeneration of cardiac cells (Rajan et al., 2014). There are hundreds of upregulated or downregulated piRNAs involved in this process, including piR-30444, piR-46538 as over-expressed, and piR-30136, piR-30368 down-expressed (Vella et al., 2016). These upregulated piRNAs silence LINE-1 and promote the differentiation of cardiac progenitor cells and the regeneration of cardiac cells by activating the AKT signalling pathway (Vella et al., 2016). Furthermore, it is evident that in the hippocampus of newborn mice, the adult neural progenitor cells (aNPCs) are rich in PIWIL2/MILI expression, and more than 570,000 putative piRNAs were identified from small RNA libraries, including one homologous to the human piR-61648. The reads of these putative piRNAs were significantly reduced, as aNPCs differentiated into neurons (Gasperini et al., 2020; Gasperini et al., 2023). Surprisingly, through *in silico* analysis, researchers discovered that repeats in 5S rRNA and tRNAs are important targets of piRNAs in aNPCs, in addition to the known targets such as mRNAs encoding ribosomal proteins, and transcripts including SINEB1 (Gasperini et al., 2020) (Figure 2D). By way of regulation, these piRNAs are anticipated to inhibit ribosome assembly, thereby reducing the rate of protein synthesis in aNPCs, and maintaining cell stemness (Gasperini et al., 2020). Moreover, the lack of MILI and piRNAs results in damage to neurogenesis, and forms reactive glia, which are commonly produced after damage to the nervous system, with its appearance often accompanied by inflammation and aging (Gasperini et al., 2023) (Table 2). Similarly, in the early *Drosophila* embryo, Aub was found to directly interact with the protein Wispy, a non-canonical poly(A) polymerase that polyadenylates the long poly(A) tails of maternal mRNAs related to reproduction in the early embryo, thereby protecting these mRNAs(Dufourt et al., 2017). The piRNA-Aub-dependent stabilization of mRNAs has already been shown to be necessary for protecting nanos in the early *Drosophila* embryo, which is considered as a germline stemness marker (Ramat and Simonelig, 2021; Rinkevich et al., 2022). Furthermore, maternal piRNA- Aub directly recruits the poly(A)-binding protein (PABP) that protects mRNA through binding to the poly(A) tail (Ramat and Simonelig, 2021). Here, Aub interacts with eIF3d, a subunit of eukaryotic initiation factor 3 complex (eIF3), initiating the translation of the target mRNAs including Nanos (Figure 3A). Nanos is closely related to the development of the germline, thereby bypassing the classic but rate-limiting protein synthesis mechanism, which is activated by eukaryotic initiation factor 4E (eIF4E) binding to the mRNA 5′cap (Ramat et al., 2020; Ramat and Simonelig, 2021).

### 2.2 piRNA-independent functions of PIWIs

**FIGURE 4:**
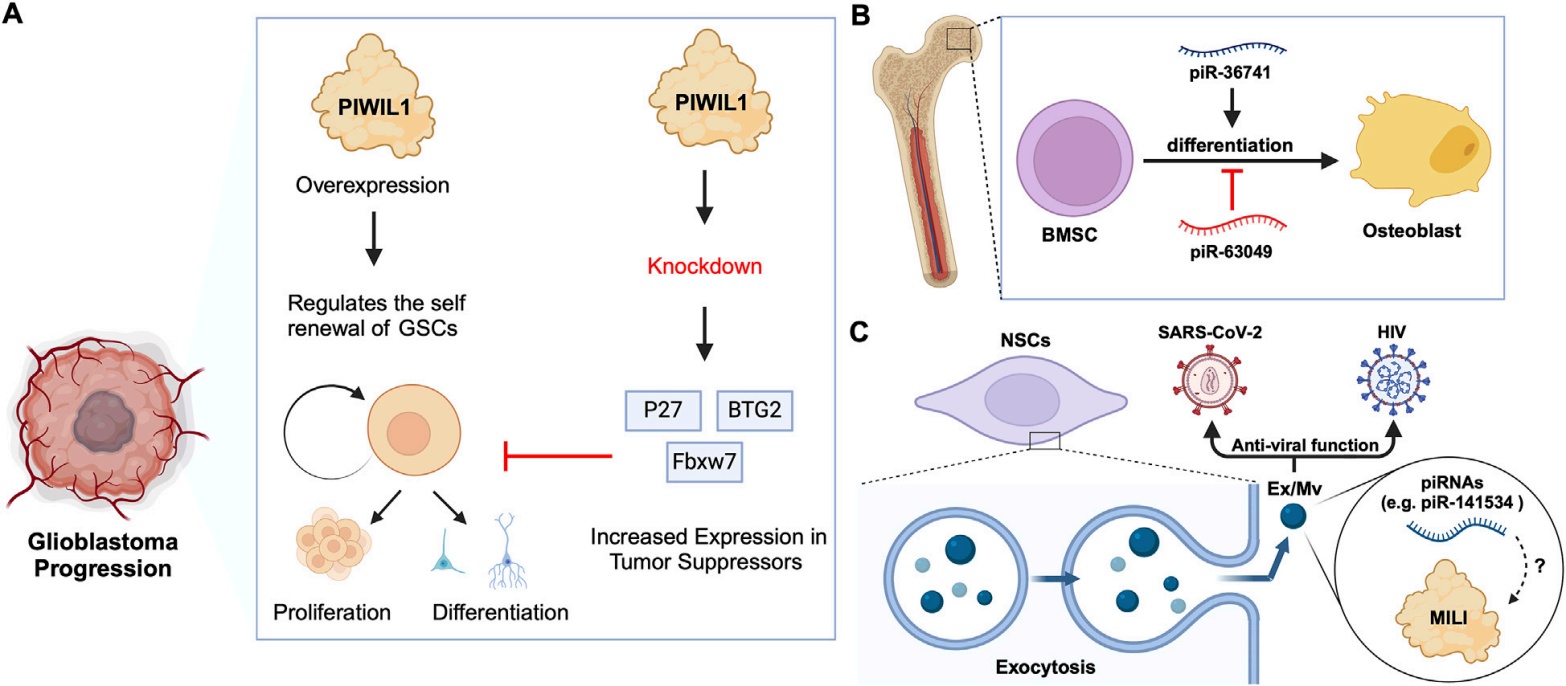
piRNA-specific and independent function of PIWIs in stem cell differentiation. **(A)** PIWIL1 overexpression in glioblastoma stem cells promotes self-renewal and tumorigenic potential. Knockdown of PIWIL1 upregulates tumor suppressors, namely BTG2 and FBXW7, inhibiting GSC self-renewal and proliferation, while promoting differentiation and senescence. **(B)** piRNAs in the differentiation of BMSCs: piR-36741 promotes the differentiation of BMSCs into osteoblasts, while piR-63049 inhibits this process. **(C)** piRNAs in exosomes/microvesicles have anti-viral roles. Mouse NSCs excrete Ex/Mvs carrying piRNAs out of the cells via exocytosis. These Ex/Mvs specific piRNAs bind and degrade target viral RNAs of HIV and SARS-Cov-2. MILI was identified as cargo in Ex/Mvs, and however, its role in the piRNA-guided antiviral functions is yet to be demonstrated (denoted as ?).

Previous studies on *Drosophila* intestinal stem cells (ISCs) show that JAK/STAT signalling can activate ISC proliferation (Ayyaz and Jasper, 2013). Under acute stress, the JAK/STAT pathway not only induces ISC proliferation but also induces the increased expression of *Drosophila* Piwi protein, thereby silencing retrotransposons and ensuring gene stability (Sousa-Victor et al., 2017; Rojas-Ríos and Simonelig, 2018). Knocking down *piwi* led to the accumulation of multiple TE transcripts, such as *Ty3* retrotransposon, *mdg1* and *hetA* (Figure 3B; Table 2), while in contrast, the loss of Aub and Ago3, which belong to the same PIWI protein family, did not affect TE silencing (Sousa-Victor et al., 2017). It is still uncertain whether the functions of these PIWI proteins depend on piRNAs(Sousa-Victor et al., 2017; Tang et al., 2023). It is worth mentioning that recent small RNA-seq data from the *Drosophila* intestine also failed to provide evidence of piRNAs’ existence (Siudeja et al., 2021). Thus, provides for the hypothesis that PIWI proteins may act alone in *Drosophila* ISCs, independent of piRNA sequences. Moreover, the STAT signalling-dependent Piwi protein activation was detected in enteroblasts, the ISC daughter cells with pluripotency, further strengthening the case for piRNA-independent PIWI function in ISCs(Sousa-Victor et al., 2017). Likewise, under disease conditions, namely in glioblastoma (GBM), PIWIL1 plays a significant role by promoting the self-renewal of glioma stem cells (GSCs). Overexpression of PIWIL1 has been consistently observed in these GSCs, contributing to their maintenance and tumorigenic potential (Huang et al., 2021). However, knockdown experiments targeting PIWIL1 have revealed a contrasting effect. The knockdown of PIWIL1 triggers an increase in tumor suppressors BTG2 and FBXW7, leading to the destabilization of the oncogene c-Myc, which in turn diminishes the proliferation and self-renewal capabilities of GSCs (Figure 4A). This effect is mediated by the enhanced stability and expression of BTG2 and FBXW7 mRNAs, which are crucial for inhibiting cell cycle progression and facilitating the degradation of oncogenic proteins like c-Myc. Consequently, reducing key stem cell factors such as Olig2 and Nestin hampers the maintenance of GSCs. These findings suggest that PIWIL1 contributes to glioblastoma growth through its regulatory effects on mRNAs. While these observations provide insights into PIWIL1’s function in mRNA regulation, further studies are needed to clarify whether these effects occur independently of piRNAs or involve indirect mechanisms (Huang et al., 2021). Also, in studying the expression of piRNA pathway genes in cancer, Genzor et al. (2019) investigated whether these genes could restore functional piRNA silencing in non-germline cells. They found that while genes like *PIWIL1, PIWIL2, PLD6* (Zucchini), and *DDX4* (VASA) were expressed in some cancer cell lines, their expression alone was not enough to form functional piRNA-PIWI silencing complexes (piRISCs). For example, in COLO205 cells, which have high levels of PIWIL1 but lack DDX4 and other key components, small RNAs bound to PIWIL1 did not show the features of true piRNAs. Instead, they resembled other RNA fragments, such as microRNAs, and did not exhibit piRNA activity (Genzor et al., 2019). Additionally, knocking down or deleting PIWIL1 did not impact transposon transcript levels, indicating that the piRNA pathway was not functional in this setting. These findings highlight the importance of careful analysis to confirm true piRNA activity and avoid misinterpreting aberrant gene expression in cancer.

### 2.3 Roles of specific piRNAs

Bone marrow stromal cells (BMSCs) include multipotent cells such as osteochondral and adipocyte progenitors (Gao et al., 2022). Through studies on BMSCs in rats and other mammals, researchers found that piRNAs are crucial for osteoblast differentiation. For instance, the piRNA, piR-63049 plays a significant role in maintaining the stemness of BMSCs(Chen et al., 2021). Meanwhile, other piRNAs, such as piR-016735, were found in exosomes derived from BMSCs, and the piRNA overexpression inhibits the differentiation of BMSCs into osteoblasts (Wang et al., 2020; Chen et al., 2021). Correspondingly, studies on human BMSCs have revealed that the expression of another piRNA, piR-36741, promotes BMSC differentiation into osteoblasts (Liu et al., 2021; Mishra et al., 2023) (Figure 4B; Table 2). Another study on human dental apical papilla stem cells (SCAP) and bone marrow mesenchymal stem cells (BMMSCs) showed that piRNAs such as piR-007832 and piR-015026 are highly expressed in BMMSCs, and the target genes of these piRNAs are enriched in pathways such as p53 signalling and the cascade associated with Epstein-Barr virus infection (Wang et al., 2020), likely involved in osteogenic differentiation (Jiang et al., 2019). Whereas, the piRNAs reported in SCAP are associated with MAPK signalling, citrate cycle, and Ras signalling pathways (Wang et al., 2020), among which, the MAPK signalling was closely related to the odonto-/osteogenic differentiation of SCAP (Wang et al., 2018). Interestingly, BMMSCs are believed to communicate with hematopoietic stem cells (HSCs) via extracellular vesicles (EVs) (Batsali et al., 2020) (Table 2). By sequencing small RNAs in human EVs, De Luca, L. *et al.* found at least two piRNAs, namely piR-020814 and piR-002732 affected the expression of numerous proteins including MPO, SLC2A5, SLAMF8, and CYP1B1(De Luca et al., 2016). These proteins have various functions such as antibacterial activity, facilitation of fructose transport, and are involved in adaptive and innate immune responses, and participate in the oxidative metabolism of xenobiotics. Likewise, induced pluripotent stem (iPS) cells from mice express at least one piRNA, piR-mmu-64162, which regulates the expression of proteins, such as Ccnd1 and Hipk1, that promote cellular reprogramming and resist cell senescence (Zhu et al., 2019). However, previous triple knockout of PIWI proteins (MILI, MIWI, MIWI2) in mouse iPS cells showed that the lack of PIWI proteins did not affect the expansion and gene expression of iPSCs(Cheng et al., 2014), raising the possibility that piRNAs function independently of PIWIs in iPSCs, which requires further studies to strengthen the argument. Similarly, murine Neural Stem Cells (NSCs) were reported to attack and neutralize viruses by releasing exosomes/microvesicles (Ex/Mv) that are enriched in approximately 150 piRNAs, including piR-mmu-141534 and piR-mmu-429488, with potential antiviral capabilities, targeting the genomes of HIV, lentiviruses and SARS-CoV-2 (Yu et al., 2020) (Figure 4C; Table 2). Furthermore, the authors of the study detected PIWIL2/MILI in the NSC Ex/Mv, however, the specific roles of this protein and how or whether it regulates specific piRNA function in attributing antiviral functions of those NSC Ex/Mv remains to be determined (Yu et al., 2020).

## 3 Emerging model systems for the study of piRNA and PIWI functions in adult/somatic stem cells

**FIGURE 5:**
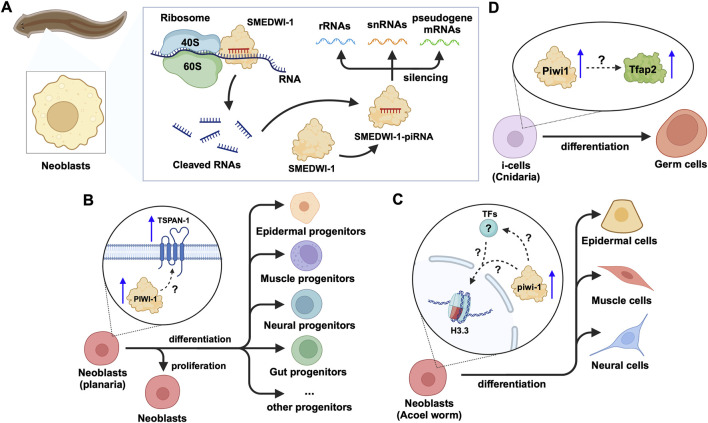
piRNA and PIWI functions in emerging models of adult stem cells in animals. **(A)** In planarian neoblasts, the SMEDWI-1-piRNA complex binds to the small ribosome subunit to cleave rRNAs, snRNAs, or pseudogene mRNAs. The cleaved RNA fragments will form new SMEDWI-1-piRNA complexes and continue to participate in target RNA cleavage. **(B)** In actively dividing neoblasts, the expression of PIWI-1 and TSPAN-1 has been detected to increase simultaneously. However, the connection between them has not been fully established in terms of their expression changes and the downstream effects on neoblast differentiation into a variety of progenitor cells. **(C)** High piwi-1 expression was detected during the differentiation of neoblasts expressing the histone variant H3.3, into epidermal, muscle and neural cells. The likely interplay between piwi-1, H3.3 and TFs involved in the process, is yet to be explored (denoted as ?). **(D)** In Cnidaria, Tfap2 and Piwi-1 jointly induce i-cells to differentiate into germ cells, with little information known in terms of how Piwi-1 controls Tfap2 expression.

In addition to the effects on mRNAs and TE transcripts, recent studies on neoblasts of the planarian *Schmidtea mediterranea* have shown that SMEDWI-1-bound piRNAs recognize and silence other non-coding RNAs, including rRNA, snRNA, and pseudogene mRNAs(Allikka Parambil et al., 2024) (Figure 5A). SMEDWI-1 was found to physically interact with eIF4A3, which is involved in the RNA unwinding, during the pioneer round of mRNA translation, suggesting the involvement of SMEDWI-1 in this process (Choe et al., 2014; Allikka Parambil et al., 2024) (Table 2). Furthermore, in planaria, Nb2 is a subtype of clonogenic neoblasts with pluripotent cell characteristics which have a high expression of PIWI-1 (Zeng et al., 2018). Here, the high levels of PIWI-1 are closely related to active cell division, as in most cases, PIWI-1 has long been considered an essential protein for the pluripotency of neoblasts (Srivastava et al., 2014; Zeng et al., 2018; Raz et al., 2021). TSPAN-1 is a surface protein on the Nb2 cell membrane that can induce stem cell mobilization and regeneration (Zeng et al., 2018). TSPAN-1+ cells exhibit pluripotency and can clone themselves to form new neoblasts, and also produce a variety of progenitor cells, such as epidermal, neural, and muscle progenitor cells (Molina and Cebrià, 2021). The TSPAN-1 protein is difficult to detect in cells at steady state, but interestingly, it is highly expressed in cells with enhanced expression of PIWI-1, which in turn is closely related to active cell division (Zeng et al., 2018; Pearson, 2022). Yet, it is still unknown how TSPAN-1 levels correlate with PIWI-1 and if any, whether PIWI-1 directly controls the abundance and/or translation of TSPAN-1 mRNA, guided or not, by specific piRNAs (Figure 5B). Similarly, the analysis of the genomic library of nemertean ribbon worm *Lineus sanguineus* identified three PIWI protein homologs, Ls-piwi1, Ls-piwi2 and Ls-piwi3, which is consistent with the data obtained from *Notospermus geniculatus* in the same phylum, Nemertea (Xu and Sun, 2020) (Table 1). Although there is no clear evidence that this organism has congenital stem cells, its somatic cell population likely contain other pluripotent cells (Zattara et al., 2019). Among the PIWIs reported in *L. sanguineus*, Ls-piwi1 is actively expressed during stem cell self-renewal, but downregulated drastically to the end of the process of differentiation, while Ls-piwi3 is only detected during posterior regeneration, and the expression of Ls-piwi2 is very weak at all stages (Xu and Sun, 2020). Moreover, the latest study on neoblasts of the acoel worm *Hofstenia miamia* showed that, in cells with high expression of a variant of histone H3 (H3.3), the expression level of piwi-1 was the highest among all neoblast subpopulations in the post-embryonic and regenerative periods (Hulett et al., 2023). This histone variant is believed to be related to the differentiation of muscle, nerve, and epidermal cells. Moreover, in neoblasts with high expression of piwi-1, differences in transcription factors (TFs) were reported to affect the direction of differentiation (Hulett et al., 2022). However, whether H3.3 or TFs directly alter the expression and/or the function of piwi-1 and its interacting partners with implications in co- or post-transcriptional gene silencing has not been elucidated (Figure 5C). Furthermore, the phylum Cnidaria includes aquatic organisms such as corals, sea anemones, jellyfish, and hydroids. One of the common adult stem cells in these organisms is called interstitial cells (i-cells). Studies on the Cnidaria *Hydractinia symbiolongicarpus* have shown that Piwi1 is active in i-cells, but its expression is reduced as i-cells differentiate into somatic cells (Varley et al., 2022). Interestingly, the i-cells expressing Piwi1 differentiate toward germ cells, after induction by Tfap2 (DuBuc et al., 2020). However, the molecular details governing this process remain unknown (Figure 5D). Further experimentation on another Cnidaria, *Hydractinia echinata* has shown that knocking down Piwi1 in i-cells impaired i-cell differentiation, thereby inhibiting the regeneration ability of *H. echinate,* after damage (Bradshaw et al., 2015; Rojas-Ríos and Simonelig, 2018). Likewise, in marine invertebrates, namely, the Ctenophore *Mnemiopsis*, researchers have found that the regions where MlPiwi1 is highly expressed, overlap with the regions with putative stem cell gene expression (Reitzel et al., 2016) (Table 1). These findings are consistent with previous reports on another Ctenophore, *Pleurobrachia*, where the piwi gene is actively expressed in regions with high mitotic activity (Alié et al., 2011). Beyond the aforementioned examples, studies on the spider *Parasteatoda tepidariorum* have shown that Pt-piwi is ubiquitously expressed in the early stages of spider embryonic development, and is essential for mitosis in early embryos, while RNAi treatment of Pt-piwi causes embryonic death (Schwager et al., 2015) (Table 1). Most embryonic tissues maintained high expression of Pt-piwi until the late embryonic stage when Pt-piwi accumulated in the formation area of primordial germ cells (PGCs) (Schwager et al., 2015) – the precise characteristic of PIWI proteins that made them a widely used potential biomarker for PGCs(Rui et al., 2020).

## 4 piRNA-dependent and independent functions of PIWIs in somatic tissues

Since the identification of piRNAs as an emerging class of small RNAs, they have substantially enriched our understanding of gene regulatory mechanisms, by way of their crucial functions, mediated by PIWI proteins, in germline processes, controlling the expression of retrotransposons (Grivna et al., 2006; Houwing et al., 2007; Loubalova et al., 2023). However, in 2015, Haifan et al. acknowledged new research exploring the roles of PIWI proteins and piRNAs in somatic cells (Ross et al., 2014). After initial investigations into the somatic role of the piRNA pathway in the ovarian cells of *Drosophila*, recent insights have revealed its functional relevance in various other tissues, such as the brain of *Drosophila* and mice (Nandi et al., 2016; Perrat et al., 2013). Additionally, a new dimension, that is, the function of PIWIs can either be dependent or independent of piRNAs, has gained traction over the years, with emerging reports constantly supporting the notion, pending in-depth validation. Thus far, the primary emphasis in somatic cell research has centred on unravelling the involvement of piRNA and PIWI proteins in diseases like cancer. However, recent studies have revealed their involvement in other human diseases such as respiratory, cardiovascular, and neurodegenerative disorders. Previously, it was believed that PIWI protein functionality relied exclusively on piRNAs. As recent research has demonstrated the non-canonical functions of PIWIs, namely their roles independent of piRNAs(Lin et al., 2020). We will provide a comparable and contrasting view of piRNA functions and PIWI proteins in TE silencing and mRNA regulation in the soma.

### 4.1 piRNA-dependent function of PIWIs

PIWI proteins form a complex with piRNAs, namely piRNA-induced silencing complex (piRISC), to mediate both transcriptional and post-transcriptional gene silencing (Loubalova et al., 2023). Not all PIWI proteins operate similarly or at the same level in the regulation of gene expression. While MILI/PIWIL2 and MIWI/PIWIL1 are involved at the post-transcriptional level, MIWI2/PIWIL4 primarily engages in transcriptional gene silencing (Loubalova et al., 2023). Here, we will specifically discuss the role of the piRNA-PIWI complexes in the regulation of mRNAs and retrotransposon silencing at the post-transcriptional level.

#### 4.1.1 mRNA regulation

Much has been established in the animal germline, where, in *C. elegans*, piRNAs target the germline transcriptome through incomplete base-pairing, thereby regulating genes transgenerationally (Wang X. et al., 2023). Also, mouse pachytene piRNAs, mostly unrelated to TE sequences, loaded onto MIWI, promote mRNA deadenylation and decay by recruiting chromatin assembly factor 1 (CAF1), and they can also direct MIWI to cleave mRNAs in a siRNA-like manner (Gou et al., 2014; Wang X. et al., 2023). Additionally, in *Drosophila* germline, specifically, in germline stem cells, it was shown that Aub/piRNAs control the expression levels of *cbl* (Rojas-Ríos et al., 2017) and dunce mRNAs(Ma et al., 2017). However, in somatic cells, piRNAs and PIWI proteins are not widely expressed and are primarily associated with various diseases, which will be discussed later in this review. In *D. melanogaster*, piRNA-loaded Aub destabilizes maternal mRNAs through endonucleolytic cleavage or by recruiting the CCR4–NOT complex consisting of exonucleases (Rouget et al., 2010; Barckmann et al., 2015; Lee et al., 2017; Wang X. et al., 2023). Further, in *C. elegans*, the PIWI protein PRG-1 is expressed in adult mechanosensory neurons and limits axon regeneration after injury, through piRNA-dependent post-transcriptional mRNA regulation. Here, PRG-1, guided by piRNAs, cleaves and represses target mRNAs including prde-1 and prg-1. PRDE-1 facilitates piRNA precursor transcription and autonomously inhibits axon regrowth in *C. elegans*, while PRG-1 uses piRNA-guided slicer activity to repress mRNAs, limiting axon regeneration and ensuring germline stability (Kim et al., 2018). Additionally, in 2018, Balaratnam et al. demonstrated the post-transcriptional regulation of the FTH1 gene by piRNAs in somatic cells (Balaratnam et al., 2018). FTH1 is elevated in a variety of cancers and has antioxidant properties (Shpyleva et al., 2011; Alkhateeb and Connor, 2013). They showed that piR-FTH1 downregulates Fth1 mRNA in triple-negative breast cancer (TNBC) cells, namely, MDA-MB-231, which indicates a potential therapeutic strategy for reducing FTH1 levels in cancer cells (Balaratnam et al., 2018). Furthermore, the study revealed that two PIWI proteins, HILI and HIWI2 are involved in mediating piR-FTH1-guided post-transcriptional regulation of the FTH1 gene. FTH1 encodes a protein which is part of the ferritin complex, responsible for iron storage and regulation in the body. It plays a crucial role in managing iron homeostasis and protecting cells from oxidative damage (Balaratnam et al., 2018).

#### 4.1.2 Retrotransposon silencing

Repetitive sequences, including TEs, constitute a significant portion of mammalian genomes and are found in nearly all organisms, including *D. melanogaster* (Curcio and Derbyshire, 2003; De Koning et al., 2011; Huang et al., 2012; Ernst et al., 2017). Retrotransposons, classified as Class I TEs, utilize a “copy and paste” mechanism, which involves RNA intermediates (Ho et al., 2024). In the germline of *D. melanogaster*, Aub and Ago3 proteins post-transcriptionally silence retrotransposon transcripts, guided by antisense piRNA sequences (Senti et al., 2015; Wang et al., 2015). Also, Watanabe et al., in 2015, revealed that piRNAs originating from pseudogenes and retrotransposons govern the expression of both functional cognate mRNA transcripts, and RNAs harbouring retrotransposon sequences. This regulatory mechanism extends to L1 retrotransposons, thereby modulating their activity, and thus safeguarding genomic stability throughout spermatogenesis (Watanabe et al., 2015). In somatic tissues, the repression of retrotransposons involves a piRNA pathway similar to the one in germ cells (Lewis et al., 2017). Briefly, cytoplasmic PIWI proteins, namely, Aub and Ago3 in *Drosophila*, and, MIWI and MILI in mice, cleave TE transcripts and help amplify piRNAs in the germline (Wang X. et al., 2023). However, nuclear PIWI proteins, specifically, Piwi in *Drosophila,* and MIWI2 in mice, bind to nascent RNAs at TE sites and recruit other proteins to silence transcription, without degrading the transcripts. Key proteins, including Maelstrom (Mael) and Panoramix (Panx), are crucial in this process (Sienski et al., 2012; Wang X. et al., 2023). Mael interacts with the Brahma complex to reduce TE transcription in somatic cells, while Panx induces transcriptional silencing and heterochromatin formation by forming complexes with Nxf2 and Nxt1 (Onishi et al., 2020). The SUMO ligase Su(var)2–10 further connects Piwi complexes to the machinery that silences chromatin, enhancing TE repression (Ninova et al., 2020b; Ninova et al., 2020a). These interactions ensure effective TE silencing in somatic tissues, demonstrating the piRNA pathway’s essential role in protecting the genome beyond germ cells.

## 5 piRNA-PIWIs in aging

**FIGURE 6:**
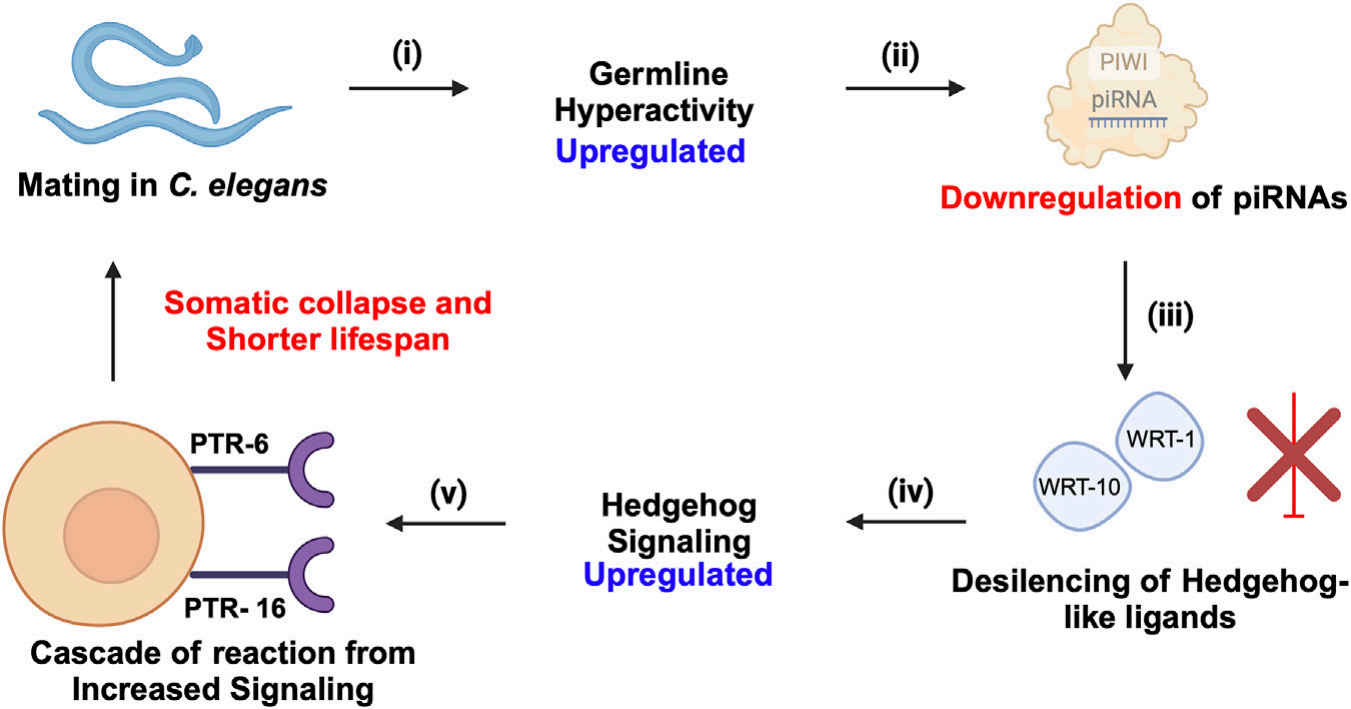
piRNA-PIWI-mediated regulation of mating-induced germline hyperactivity and somatic collapse in the aging of *C. elegans*. (i) Mating initiates hyperactivity in the germline. (ii) This germline hyperactivity leads to the downregulation of piRNAs. (iii) The downregulation of piRNAs results in the de-silencing and activation of Hedgehog-like ligands, such as wrt-1 and wrt-10. (iv) The activation of these Hedgehog-like ligands enhances Hedgehog signalling, which impacts somatic cells through specific receptors, PTR-6 and PTR-16. (v) The increased Hedgehog signalling in somatic cells triggers a cascade of events leading to somatic collapse, characterized by a decline in somatic cell function and health, ultimately accelerating aging and reducing lifespan.

The role of piRNA-PIWIs in the aging process has not been widely discussed. Recent insights in *C. elegans* have highlighted that transposable elements (TEs) contribute significantly to aging by promoting genomic instability, with their activity increasing as the organism ages due to epigenetic changes, such as the accumulation of N6-adenine methylation at active TE loci (Sturm et al., 2023). The piRNA- PIWI pathway, essential in germline cells for silencing TEs, was shown to enhance lifespan when ectopically expressed in somatic cells, likely by suppressing TEs and reducing genomic instability. Downregulation of active TEs, such as *Tc1* and *Tc3,* extended lifespan in *C. elegans*, while elevated TE activity was associated with somatic decline, supporting a direct causal link between TE mobilization and aging (Sturm et al., 2023). Moreover, these findings align with recent insights that piRNA-PIWI influences longevity by modulating Hedgehog signalling after mating-induced germline hyperactivity (Shi and Murphy, 2023). The downregulation of piRNAs leads to the activation of Hedgehog-like ligands, which communicate germline status to somatic cells, resulting in somatic collapse and reduced lifespan (Shi and Murphy, 2023) (Figure 6). Wet age-related macular degeneration (wAMD) is a major cause of blindness in older adults in industrialized countries (Muraleva et al., 2019). PIWIL4, also referred to as HIWI2 in humans and MIWI2 in mice is involved in choroidal neovascularization (CNV), suggesting a critical role for piRNA-PIWIs in the pathological processes underlying wAMD (Ren et al., 2023). It plays a key role by controlling VEGF-driven angiogenesis through its interaction with specific piRNAs. Studies using laser-induced CNV models in mice showed increased PIWIL4 expression in retinal pigment epithelium (RPE)-choroid-sclera complexes and choroidal endothelial cells (MCECs). Experimental evidence demonstrated that reducing PIWIL4 levels with shRNA lentiviral and adeno-associated virus (AAV) systems lowered CNV lesion areas and reduced abnormal blood vessel growth *in vivo*. *In vitro* silencing of PIWIL4 blocked VEGF-induced cell growth, migration, and tube formation, while also lowering VEGF secretion and VEGFR2 activity, showing its importance in VEGF-related pathways. High-throughput sequencing identified several piRNAs, including piR-mmu-72603, piR-mmu-671578, and piR-mmu-7577,132, as highly expressed in CNV tissues (Ren et al., 2023). These piRNAs, likely working with PIWIL4, were linked to blood vessel growth and repair through biological pathway analyses. The co-localization of PIWIL4 with areas of neovascularization further highlights its role in abnormal blood vessel growth. Together, these findings suggest that the PIWIL4-piRNA system is central to the development of wAMD and may serve as a target for new treatments. Additionally, the presence of piRNAs in eye fluids suggests their potential as non-invasive biomarkers, opening new possibilities for precise diagnosis and treatment of wAMD (Ren et al., 2023).

## 6 piRNAs and PIWI proteins in human diseases

The independent roles of PIWI proteins outside their traditional association with piRNAs have been explored mainly in the context of human diseases. The expression and function of PIWIs and potential piRNAs along with the downstream targets responsible for the disease phenotype are detailed in Table 3.

**TABLE 3:** PIWI proteins, their expression and function in human diseases.

PIWI proteins	Expression	Disease	Function	References
PIWIL1	Upregulated	Amyotrophic Lateral Sclerosis (ALS)	Dysregulated in ALS	Hardiman et al. (2017), Abdelhamid et al. (2022)
	Upregulated	Alzheimer’s Disease (AD)	Increased expression in AD hippocampal neurons, linked to tau pathology-induced chromatin changes	Sato et al. (2023), Masters et al. (2015)
	N.D.	Gastric Cancer	Recruits NMD machinery, leading to downregulation of tumor suppressor mRNA such as VCL, LAMC3, FLNA, MYO18B, SRCIN1, and TPM2 and promoting cancer progression	Lin et al. (2020)
		Hepatocellular Carcinoma (HCC)	Associated with HCC progression; interaction with piR-017724 is also implicated in HCC development	Wu et al. (2023)
		Pancreatic Cancer	Interaction with piR-017061 inhibits EFNA5 mRNA; downregulation of PIWIL1 upregulates EFNA5, influencing cancer progression. Exhibits both detrimental and protective roles	Xie et al. (2021)
PIWIL2	Upregulated	Lung Cancer	Drives progression of non-small cell lung cancer through induction of CDK2 and Cyclin A expression	Qu et al. (2015)
	Downregulated	Lung injuries (linked to BaP exposure)	Drives progression of non-small cell lung cancer through induction of CDK2 and Cyclin A expression	Lei et al. (2024)
	Upregulated	Cardiomyocyte necroptosis	Regulates necroptosis in cardiomyocytes, potentially interacting with the piRNA, namely HNEAP.	Wang et al. (2023a)
PIWIL3	No expression observed	Amyotrophic Lateral Sclerosis (ALS)	No function	Abdelhamid et al. (2022)
PIWIL4	Downregulated			
	Upregulated	Cardiomyocyte necroptosis	Inhibits DNMT1-mediated methylation of Atf7 mRNA, increasing ATF7 expression and suppressing CHMP2A	Wang et al. (2023a)

N.D., not determined.

### 6.1 Cancer

**FIGURE 7:**
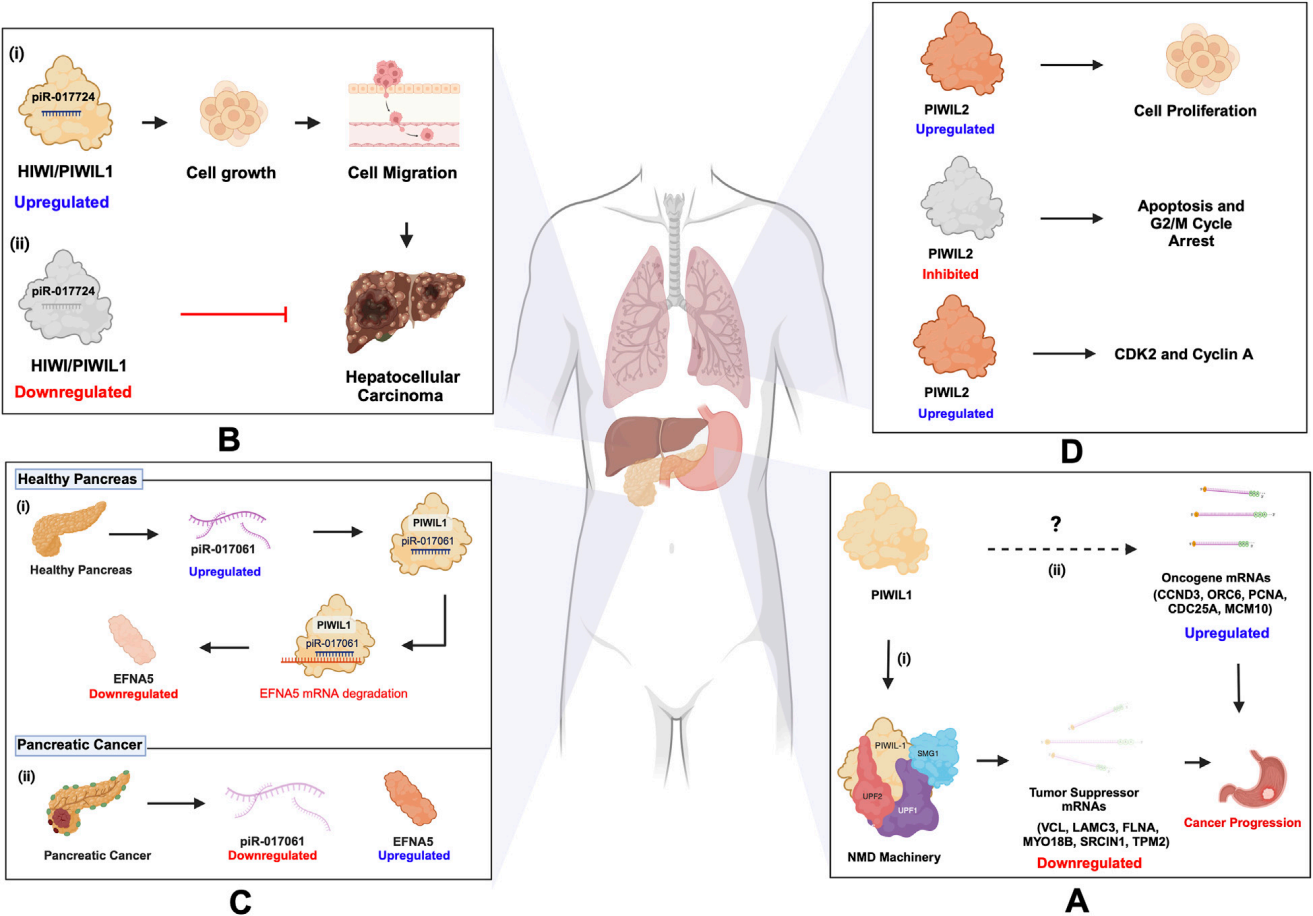
piRNA and PIWI functions in cancer. **(A)** (i) PIWIL1 downregulates tumor suppressor mRNAs (VCL, LAMC3, FLNA, MYO18B, SRCIN1, TPM2) by recruiting the NMD machinery (SMG1, UPF2, UPF1), promoting gastric cancer progression. (ii) PIWIL1’s role in upregulating oncogene mRNAs (CCND3, ORC6, PCNA, CDC25A, MCM10) remains unexplored. **(B)** (i) PIWIL1/HIWI upregulation, along with piR-017724, is associated with the progression of hepatocellular carcinoma (HCC). (ii) Downregulation of PIWIL1/HIWI along with piR-017724 prevents the development of HCC. **(C)** PIWIL1/HIWI in pancreatic cancer: (i) The upregulation of piR-017061, which binds with PIWIL1, inhibits the mRNA coding for EFNA5. (ii) Conversely, the downregulation of piR-017061 resulted in the increased expression of EFNA5, an oncogene implicated in the progression of pancreatic cancer. **(D)** PIWIL2 in lung cancer: PIWIL2 induces the expression of CDK2 and Cyclin A, which inhibit apoptosis and prevent G2/M cycle arrest, thus progressing NSCLC. Inhibition of PIWIL2 leads to apoptosis and G2/M cycle arrest.

Cancer remains one of the most prevalent and challenging health concerns globally, despite extensive research efforts over the decades (Sung et al., 2021). While significant progress has been made in understanding cancer biology, there are still many gaps in our knowledge that warrant further exploration. Amidst the heterogeneity of cancer, emerging evidence has highlighted the involvement of piRNAs and their associated PIWI proteins in the development and progression of various cancer types, including those affecting the stomach, lungs, pancreas, and liver (Liu et al., 2019). The multifaceted roles of piRNAs and PIWI proteins in cancer underscore the need for a comprehensive understanding of their mechanisms and potential as biomarkers and targets (Ameli Mojarad et al., 2022). Recent findings indicate that PIWI protein involvement in cancer can either depend on piRNA or function independently. Similarly, piRNAs can either partner with PIWI proteins or act autonomously within cancer pathways (Zhang et al., 2023). In 2020, over one million cases of stomach-related cancer were recorded, resulting in over 700 thousand reported fatalities (Sung et al., 2021). In the same year, Haifan et al. explored the independent function of PIWIL1/HIWI in gastric cancer (Lin et al., 2020), and demonstrated that PIWIL1 recruits the NMD (nonsense-mediated mRNA decay) machinery, a cellular pathway that degrades aberrant or selective mRNAs to prevent harmful protein production, involving components like SMG1, UPF2, and UPF1. This recruitment leads to the downregulation of tumor suppressor mRNAs, such as *VCL, LAMC3, FLNA, MYO18B*, *SRCIN1*, and *TPM2*, which are associated with cell adhesion and migration, facilitating gastric cancer progression. (Figure 7A; Table 3). However, PIWIL1 role in the upregulation of oncogene mRNAs such as *CCND3, ORC6, PCNA, CDC25A*, and *MCM10* remains unexplored (Lin et al., 2020; Ameli Mojarad et al., 2022). Additionally, piR-1245 is in high concentration in gastric juice from gastric cancer patients, compared to healthy individuals, suggesting its potential as a biomarker (Zhou et al., 2020; Ameli Mojarad et al., 2022). Likewise, the upregulation of PIWIL1 was reported in Hepatocellular Carcinoma (HCC) (Xie et al., 2015; Xu et al., 2020). In 2023, Wu et al. revealed that PIWIL1’s interaction with piR-017724 was implicated in HCC development (Figure 7B; Table 3) (Wu et al., 2023). Interestingly, a paradoxical function of PIWIL1/HIWI has surfaced, where the upregulation of piR-017061, which binds with PIWIL1, inhibited the mRNA translation of Ephrin A5 (EFNA5), involved in regulating cell growth and proliferation in pancreatic cancer. Elevated levels of EFNA5 markedly enhance the growth of pancreatic cancer cells, whereas reducing its expression leads to diminished cell growth. These findings suggest that EFNA5 functions as an oncogene in the context of pancreatic cancer. Conversely, the downregulation of piR-017061 was shown to upregulate EFNA5, an oncogene in pancreatic cancer progression (Xie et al., 2021) (Figure 7C; Table 3). Thus, demonstrating that HIWI exhibits a multifaceted role, exerting a detrimental influence on cancer progression depending on the cell type, while concurrently fostering tissue health, a duality that underscores its complexity and importance in the regulation of cell development. On the other hand, PIWIL2/HILI has emerged as a pivotal player in lung cancer, the second most prevalent cancer worldwide, according to the 2020 statistics (Sung et al., 2021; Jian et al., 2022). PIWIL2 drives the progression of non-small cell lung cancer through the induction of CDK2 and Cyclin A expression, thereby inhibiting apoptosis, and G2/M cycle arrest (Qu et al., 2015) (Figure 7D; Table 3). Benzo(a)pyrene (BaP), classified as a carcinogen is linked to various forms of lung injuries, particularly lung cancer, through exposure from sources such as cigarette smoke and pollution from fine particles in the air (measured as PM_2.5_). Recent epidemiological studies revealed that PM_2.5_ is involved in the suppression of three piRNAs (piR-004153, piR-020326 and piR-020388), which in turn promoted lung injuries associated with BaP exposure (Chen et al., 2019; Lei et al., 2024). Recent studies have emphasized the importance of rigorous annotation when studying piRNAs in somatic cells, as misannotations can lead to misleading conclusions. Tosar et al. (2021) pointed out that SNORD57, a small nucleolar RNA, is often misidentified as piR-54265, previously proposed as a biomarker for colorectal cancer. Their findings stress that SNORD57, rather than piR-54265, is a more reliable biomarker, highlighting the need for careful evaluation of piRNA data in somatic research (Tosar et al., 2021). This underscores the complexity of distinguishing *bona fide* piRNAs from other small RNAs in somatic tissues and the importance of robust *in silico* analysis for accurate identification. Such methodological precision is critical for understanding the diverse roles of piRNAs in diseases, as their functions extend beyond canonical germline processes to influence mRNA regulation and transposon silencing in somatic contexts.

### 6.2 Cardiovascular diseases

**FIGURE 8:**
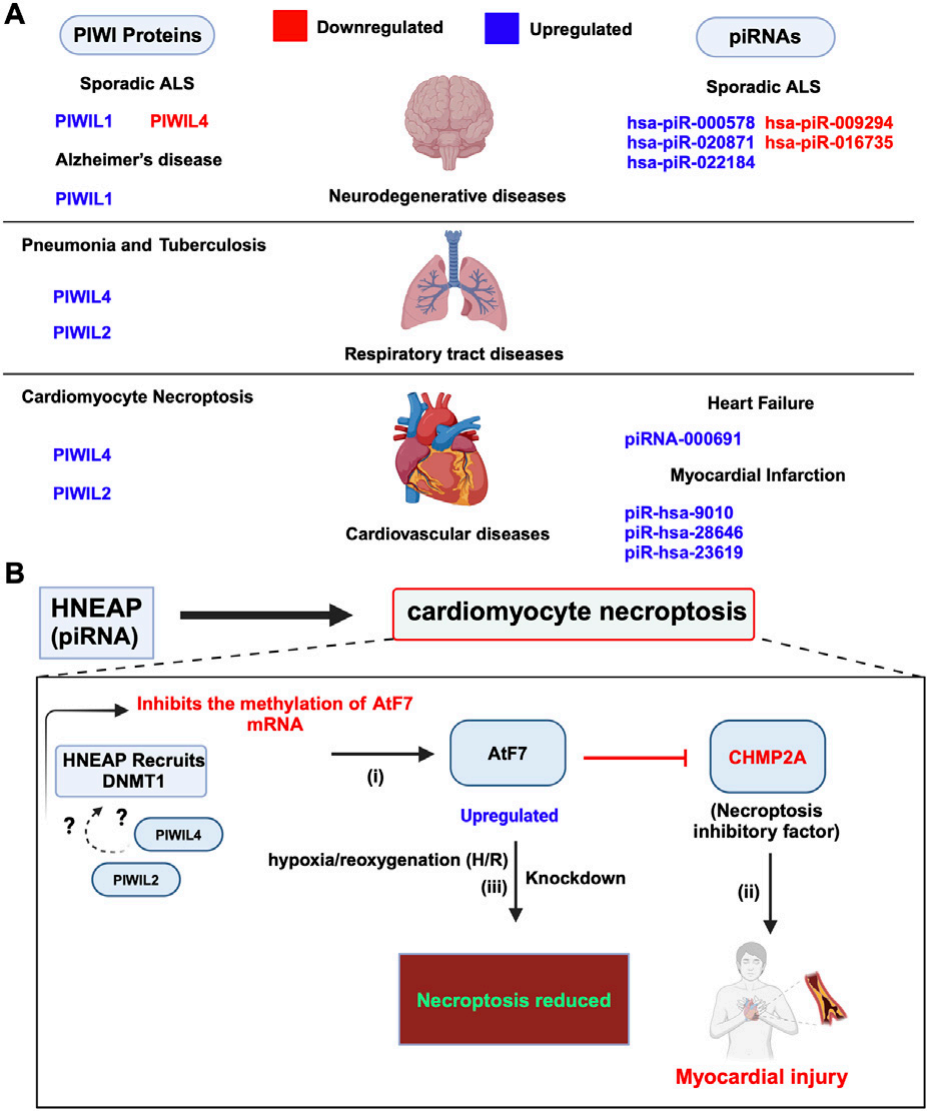
Dysregulated expression and functions of piRNA and PIWIs in other human diseases. **(A)** Dysregulated piRNAs and PIWI proteins in cardiovascular, neurodegenerative and respiratory tract diseases. **(B)** piRNA HNEAP in cardiomyocyte necroptosis: (i) HNEAP recruits DNMT1 to Atf7 mRNA, inhibiting its m^5^C methylation, which results in increased Atf7 mRNA transcription and protein expression. Whether HNEAP forms a complex with PIWIL2 and PIWIL4 is yet to be determined (denoted as ?). (ii) Elevated Atf7 expression inhibits the transcription of CHMP2A, an anti-necroptotic factor, leading to increased cardiomyocyte necroptosis. (iii) Knockdown of Atf7 reduces cardiomyocyte necroptosis induced by pathological stimuli, such as hypoxia/reoxygenation (H/R) exposure., indicating Atf7’s role as a pro-necroptotic transcription factor.

Cardiovascular disease (CVD) causes around 18.6 million deaths annually worldwide and encompasses myocardial issues such as hypertrophy, infarction, and atherosclerosis, aggravated by risk factors including hyperlipidemia, hypertension, and obesity, particularly prevalent in type 2 diabetes (Liu et al., 2019). In recent years, there has been growing interest in exploring the role of piRNAs concerning these cardiovascular conditions. Research studies thus far mainly focused on identifying piRNAs as biomarkers for these diseases. For instance, recently, a novel aortic valve calcification-associated piRNA has been identified, called AVCAPIR which increases valvular calcification and promotes the progression of calcific aortic valve disease (CAVD) (Han et al., 2024). Additionally, a highly upregulated piRNA, namely piRNA-000691, known as CFRPi, is reported to be abundant in the heart and is expressed specifically in cardiac fibroblasts, promoting cardiac fibrosis under chronic pressure overload, thereby contributing to heart failure (Chen et al., 2024). Moreover, the piRNAs, namely, piR-hsa-9010, piR-hsa-28646, and piR-hsa-23619 are significantly upregulated in acute myocardial infarction (AMI) patients, with piR-hsa-28646 and piR-hsa-23619 showing higher expression in Primary Human Umbilical Vein Endothelial Cells (HUVEC) (Figure 8A), with involvement in key signalling pathways such as Wnt and TNF signalling. Wnt pathway is activated in endothelial cells of the infarct area and plays a role in angiogenesis following myocardial infarction. The TNF pathway is crucial in mediating the inflammatory response and affecting vascular function after myocardial infarction (Huang et al., 2023). Despite these intriguing findings, there is a lack of studies on the expression and function of PIWI proteins in mammalian model systems including mice, which is crucial for understanding the implications of the piRNA pathway in cardiac development. However, A recent study has highlighted potential interactions between PIWI proteins, including PIWIL4 and PIWIL2, with a newly identified piRNA known as Heart Necroptosis-Associated piRNA (HNEAP). In 2023, Wang et al. demonstrated that HNEAP plays a crucial role in regulating cardiomyocyte necroptosis by influencing the 5-methylcytosine (m^5^C) modification of the mRNA of Activating Transcription Factor 7 (Atf7). Specifically, HNEAP inhibits the DNA (Cytosine-5)-Methyltransferase 1 (DNMT1)-mediated methylation of Atf7 mRNA. This inhibition leads to increased ATF7 expression, which in turn suppresses CHMP2A, a key anti-necroptotic factor, highlighting HNEAP’s significance in cardiac pathophysiology (Wang K. et al., 2023) (Figure 8B; Table 3). Notably, HNEAP exhibits specific enrichment in heart tissue and cardiomyocytes, suggesting potential interactions with PIWI proteins, namely PIWIL4 and PIWIL2 (Wang K. et al., 2023). These findings illuminate novel mechanisms of piRNA-PIWI-mediated post-transcriptional gene regulation and propose HNEAP as a promising therapeutic target for addressing ischemic heart injury (Wang K. et al., 2023).

### 6.3 Neurodegenerative diseases

piRNAs and PIWI proteins are implicated in different neurodegenerative diseases, including Parkinson’s, Alzheimer’s, and Amyotrophic lateral sclerosis (ALS) (Sato et al., 2023). In ALS, there is a loss of motor neurons in the brain and spinal cord, leading to muscle weakness, paralysis, and eventually death (Hardiman et al., 2017). In 2022, the study conducted by Abdelhamid et al. found that, in sporadic ALS samples, three piRNAs (hsa-piR-000578, hsa-piR-020871, and hsa-piR-022184) were upregulated, while two (hsa-piR-009294 and hsa-piR-016735) were downregulated (Abdelhamid et al., 2022). They also observed dysregulation of PIWI proteins: PIWIL4 was downregulated, while PIWIL1 was upregulated, and PIWIL2 and PIWIL3 were not detectable in the samples (Figure 8A; Table 3) (Abdelhamid et al., 2022). These findings suggest the potential of piRNAs as biomarkers for treatment options, where, additional research is required to substantiate this claim. Similarly, Alzheimer’s is the most common of all the other neurodegenerative diseases followed by Parkinson’s, and is characterized by memory loss and cognitive decline (Masters et al., 2015; Bloem et al., 2021). In Alzheimer’s disease (AD), dysregulated piRNAs have been identified as potential biomarkers and therapeutic targets. Jain et al. (2019) found altered levels of piR-019324, piR-019949, and piR-020364 in cerebrospinal fluid (CSF) exosomes of AD patients. Roy et al. (2017) observed the upregulation of piRNAs in AD brains, correlating with AD risk single nucleotide polymorphisms (SNPs). Additionally, increased expression of HIWI/PIWIL1 was noted in AD hippocampal neurons, possibly linked to tau pathology-induced chromatin changes. These findings highlight the role of piRNAs and PIWI proteins in AD pathogenesis (Roy et al., 2017; Jain et al., 2019; Sato et al., 2023). In contrast, the role of piRNAs and PIWI proteins in Parkinson’s disease (PD) remains largely unexplored, with limited findings reported in the literature. According to the review by Sato et al. (2023), recent studies, such as those by Zhang and Wong (2022), have begun to shed light on this area. They explored the role of piRNAs in PD where they identified over 200 piRNAs in the prefrontal cortex and over 500 piRNAs in the amygdala, a region often affected by Lewy bodies (Zhang and Wong, 2022). Among these, more than 15 piRNAs in the prefrontal cortex, and over 50 in the amygdala showed differential expression in PD patients. Notably, those 50 piRNAs differentially expressed in the amygdala were predicted to target over 20 protein-coding genes, including those critical for mitochondrial function, such as MT-CO1 and MT-CO3, involved in the onset of PD (Zhang and Wong, 2022; Sato et al., 2023).

### 6.4 Respiratory tract diseases

The roles of piRNAs and PIWI proteins have been noted in a spectrum of respiratory tract diseases, including pneumonia, asthma, tuberculosis, pulmonary arterial hypertension, and interstitial lung disease (ILD) (Yao et al., 2023). In pneumonia, PIWIL2 and PIWIL4 are upregulated in response to endoplasmic reticulum (ER) stress, and MIWI2 modulates immune responses in multiciliated airway epithelial cells in mice (Figure 8A) (Hogan et al., 2014; Wasserman et al., 2017; Gebert et al., 2018). PIWIL2 in ILD, interacts with heat shock protein 90 (Hsp90) to modulate TGF-β signalling and fibrosis (Zou et al., 2020). In tuberculosis (TB), the increased activity of PIWIL2 and PIWIL4, along with the significant presence of piRNAs such as piRNA-1007467, piR-hsa-1344, and piR-hsa-1944, highlights the complex involvement of the piRNA-PIWI axis in TB development. These piRNAs are implicated in influencing disease progression and modulating the host immune response (Zhang et al., 2019). We believe future studies on the mechanisms underlying the changes in PIWI expression and the targets, namely protein-coding genes might shed light on the pivotal information necessary for developing accurate and effective piRNA-PIWI-based treatment strategies for ILD and TB.

## 7 Conclusion and perspectives

Studies over the past few years have expanded our understanding of piRNAs and PIWI proteins beyond their traditional roles in the animal germline, revealing the significance of this pathway in the soma. Initially identified for their roles in controlling TEs in germ cells, piRNA-PIWIs are now recognized for their functions in mRNA regulation, translation, and retrotransposon expression in adult stem cells, somatic tissues, and human diseases. For instance, the ping-pong cycle associated with piRNA amplification and TE transcript silencing is ubiquitous in stem cells of *Drosophila*, humans, mice, and planarians (Ernst et al., 2017; Huang and Wong, 2021). The piRNA-PIWI complex controls the localization and cleavage of histone mRNAs in planarian neoblasts and can silence ribosome-related RNAs in mouse aNPCs(Rouhana et al., 2014; Gasperini et al., 2020; Allikka Parambil et al., 2024). Additionally, PIWIs can function independently of piRNAs in stem cells, e.g. Piwi in *Drosophila* ISCs silences TE transcripts (Sousa-Victor et al., 2017; Siudeja et al., 2021), and PIWI-interacting partners such as SMEDWI-1 participate in the pioneer round of translation via interaction with eIF4A3 in planarian neoblasts (Choe et al., 2014; Allikka Parambil et al., 2024). Despite these interesting observations, it is still unclear whether the functional outcome of piRNA-PIWIs manifests the unique characteristics of stem cells, and there is a general lack of mechanistic insights from these studies, which is a potential direction for future research expanding on the non-canonical roles of piRNA-PIWIs in stem cells. Particularly, in the case of piRNA-independent roles of PIWIs, questions remain unanswered as to whether and how PIWI proteins (unaccompanied by piRNAs) recognize and (directly) bind target transcripts or do they recruit interacting partners, namely RNA-binding proteins (RBPs), for selecting the targets. To address this knowledge gap, we require an in-depth analysis of the target repertoire of PIWI proteins and their homologs in different organisms. This would empower us to dissect the biological significance of these target RNA-PIWI associations in stem cell pluripotency and differentiation. We reckon that such a comprehensive set of data is feasible with the latest, highly sensitive *in situ* workflows, such as INSCRIBE which can capture endogenous RNA-protein interactions in primary tissues (Liang et al., 2024). This technique is particularly beneficial for dealing with precious tissues including planarian neoblasts and mouse aNPCs. Moreover, findings from these high-throughput data sets will likely aid in understanding genuine RBP-RNA interactions underlying the development of piRNA-related human diseases. In contrast to the studies emphasizing the importance of piRNA and/or PIWIs in stem cells, the study by Cheng EC et al. revealed that the triple knockout of PIWIs had a modest or non-significant effect in terms of cellular expansion and the underlying gene expression in mouse iPSCs(Cheng et al., 2014). These results raise the question of whether PIWIs and thereby piRNAs are required for stem cell development. Given the unique nature of iPSCs, unlike the naturally present neoblasts or other adult stem cells in different animal species, the piRNA pathway may be dispensable in this specific context. Alternatively, the treatment of cells with the Yamanaka factors (Cerneckis et al., 2024) to generate iPSCs might have countered the effects of the piRNA pathway or shifted the balance in favour of other small RNAs (e.g. miRNAs) to compensate for piRNA-PIWI contributions to maintaining gene expression in these cells.

Since their discovery in *Drosophila*, the majority of work on the piRNA pathway has been conducted in classical animal models, including *C. elegans*, and to some extent, in mice (Ernst et al., 2017; Kim et al., 2018; Huang and Wong, 2021; Ramat and Simonelig, 2021). Since piRNA sequence conservation is low across species (Parhad and Theurkauf, 2019), unlike other small RNAs, such as miRNAs, it is conceivable that piRNA function in gene silencing varies across model systems that facilitate the study of stem cell development and the pathogenesis of human diseases. These variations underscore the need to explore this pathway critically to system specificity and whether common features exist between experimental systems that could be closely applied to human biology and pathology. To aid in such understanding, it is worthwhile analyzing sequence similarities, if any, among piRNAs between systems that control the binding pattern of PIWIs and/or their interacting partners to these small RNAs and the target transcripts. Insights from these analyses might shed some light on the underlying causes of piRNA-independent functions of PIWIs, where stage and tissue-dependent expression of piRNA is minimal or null, prompting PIWIs to go solo owing to their interaction with other RBPs or high affinity for specific motifs in target transcripts, to deliver gene silencing. Despite these knowledge gaps surrounding the peculiar functions of the piRNA pathway, the growing body of evidence indicates that PIWI proteins can operate independently of piRNAs, especially in diseases like cancer, where they regulate oncogenic pathways through mRNA degradation and translational inhibition. This dual functionality underscores the versatility of PIWI proteins in diverse cellular processes and disease pathogenesis. Thus targeting the piRNA pathway has become a promising avenue for therapeutic interventions. But it would only be possible from a better understanding of i) the precise mechanisms underlying PIWI protein interactions with other cellular components during the development of tumors, ii) the molecular details of PIWI-independent functions in the development of tissue-specific cancers, and iii) the stage-dependent expression of specific piRNAs and/or PIWIs and their targets in the healthy vs. malignant/transformed tissues or cells.

The involvement of the piRNA pathway in age-related diseases, such as neurodegenerative disorders and cardiovascular conditions, is an emerging trend that underscores the broader relevance of this pathway in human health. Clinical studies have identified specific piRNAs as potential biomarkers for cardiovascular and neurodegenerative disorders, indicating their diagnostic utility and therapeutic potential (Abdelhamid et al., 2022; Sato et al., 2023; Chen et al., 2024; Han et al., 2024). Understanding how piRNAs contribute to the molecular mechanisms underlying these diseases could lead to novel therapeutic approaches aimed at mitigating the effects of aging and promoting healthy longevity. Despite the advancements in these disease contexts, several gaps persist in our comprehensive understanding of the piRNA-PIWI axis. The interaction between piRNAs and other small RNAs, such as miRNAs and siRNAs, in the regulation of gene expression, is not well characterized in somatic cells. The potential crosstalk between these pathways could reveal new layers of regulatory complexity, with implications for understanding cellular homeostasis and disease states. The variability in PIWI protein functions across species and cell types complicates the translation of findings from model organisms to humans, as well as within and across the tissue/cell types. Addressing these issues will require a concerted effort to standardize methodologies and establish cross-species comparisons, revealing that these protein functions are regulated similarly across various cell types or differ significantly.

In conclusion, the piRNA-PIWI pathway represents a versatile and essential regulatory system with broad implications for stem cell biology, somatic cell function, and disease. Its roles in processes ranging from transposon silencing to mRNA regulation, and its emerging significance in somatic tissues, underscore the need for continued research. Understanding these mechanisms will be crucial for developing targeted therapies that harness the regulatory potential of the piRNA-PIWI pathway, offering new hope for treating a range of conditions, from cancer to neurodegenerative and cardiovascular diseases.
